# Transcriptomic Response Dynamics of Human Primary and Immortalized Adrenocortical Cells to Steroidogenic Stimuli

**DOI:** 10.3390/cells10092376

**Published:** 2021-09-09

**Authors:** Kimberly Wellman, Rui Fu, Amber Baldwin, Juilee Rege, Elisabeth Murphy, William E. Rainey, Neelanjan Mukherjee

**Affiliations:** 1 RNA Bioscience Initiative, University of Colorado School of Medicine, Aurora, CO 80045, USA; kimberly.wellman@cuanschutz.edu (K.W.); rui.fu@cuanschutz.edu (R.F.); amber.baldwin@cuanschutz.edu (A.B.); elisabeth.murphy@cuanschutz.edu (E.M.); 2 Department of Biochemistry and Molecular Genetics, University of Colorado School of Medicine, Aurora, CO 80045, USA; 3 Department of Molecular and Integrative Physiology, University of Michigan School of Medicine, Ann Arbor, MI 48109, USA; juilee@med.umich.edu (J.R.); wer@med.umich.edu (W.E.R.)

**Keywords:** adrenal, steroid hormones, stimulus response, ACTH, angiotensin II

## Abstract

Adrenal steroid hormone production is a dynamic process stimulated by adrenocorticotropic hormone (ACTH) and angiotensin II (AngII). These ligands initialize a rapid and robust gene expression response required for steroidogenesis. Here, we compare the predominant human immortalized cell line model, H295R cell, with primary cultures of adult adrenocortical cells derived from human kidney donors. We performed temporally resolved RNA-seq on primary cells stimulated with either ACTH or AngII at multiple time points. The magnitude of the expression dynamics elicited by ACTH was greater than AngII in primary cells. This is likely due to the larger population of adrenocortical cells that are responsive to ACTH. The dynamics of stimulus-induced expression in H295R cells are mostly recapitulated in primary cells. However, there are some expression responses in primary cells absent in H295R cells. These data are a resource for the endocrine community and will help researchers determine whether H295R is an appropriate model for the specific aspect of steroidogenesis that they are studying.

## 1. Introduction

Steroid hormones signal a coordinated biological response essential to homeostasis. In the adrenal gland, the cortex produces the three major steroid hormone classes, each with distinct roles: the mineralocorticoid aldosterone is the master regulator of blood pressure, the glucocorticoid cortisol is important for the stress response, and androgens determine sex characteristics [1,2]. The production of these bioactive molecules is highly consequential for human health. For example, primary aldosteronism is caused by the overproduction of aldosterone and leads to hypertension [3,4,5]. Likewise, overproduction of cortisol causes Cushing’s syndrome, which leads to severe metabolic disorders [6]. A detailed understanding of the regulation of steroidogenesis is imperative to harness these mechanisms for future treatments. 

Synthesis of steroid hormones is induced by stimuli and its steps are complex. In the simplest terms, free cholesterol is modified by a series of enzymes leading to steroid hormone products that are secreted from the cell. Adrenocorticotropic hormone (ACTH) and angiotensin II (AngII) are key physiological ligands that act on the adrenal cortex to stimulate steroidogenesis [7,8,9]. ACTH binds to the melanocortin 2 receptor (*MC2R*) and results in an activation of adenylyl cyclase and the cAMP-PKA-CREB signal transduction cascade. AngII is the main secretagogue for cells of the *zona glomerulosa* and binds the type I angiotensin II receptor [10]. Activation through this receptor increases intracellular calcium in a G protein-dependent manner and stimulates MAPKs. In cells of the *zona fasciculata*, activation of these pathways results in transcriptional upregulation of pro-steroidogenic genes [2,11,12]. These induced mRNAs encode proteins regulating cholesterol transport and steroid biosynthesis, such as steroidogenic acute regulatory protein (*STAR*), 11β-hydroxylase (*CYP11B1*), and aldosterone synthase (*CYP11B2*).

The predominant research model for studying molecular mechanisms regulating human adrenocortical steroidogenesis is the immortalized H295R cell line. This adrenocortical carcinoma cell line was originally isolated from a human tumor and underwent selection for adherence [13,14,15,16]. The defining characteristic of the H295R line is the production of aldosterone and cortisol in response to steroidogenic stimuli. H295R cells are not representative of a single cortical cell type and are thought to be de-differentiated, thus capable of producing both hormones [16]. Since H295R cells are minimally responsive to ACTH [17], forskolin is used in its place as it bypasses the *MC2R* receptor and activates adenylyl cyclase. There has been some effort to compare this carcinoma cell line to normal adrenal tissue, but the comparisons are largely biochemical [18,19,20,21]. Primary adrenocortical cells cultured from healthy tissue have also been used to study stimuli-induced adrenocortical steroidogenesis [22]. However, these cells are used less frequently because they require access to human tissue, specialized expertise in primary cell culture, and can only be maintained for a limited number of cell divisions. While both primary and H295R cells have been impactful for steroidogenesis research, it is important to directly compare the two to understand the limitations and strengths of each model. 

Much of the work on human steroidogenesis relies heavily on end point measurements of hormone levels [21,23,24]. These assays are informative for detecting differences in hormone levels dependent on an experimental condition. However, steroidogenesis involves interdependent molecular pathways that rely on multiple levels of gene regulation. Previous studies in H295R have delineated the temporal coordination of gene expression in response to AngII crucial for eliciting the appropriate production of aldosterone [25]. Unfortunately, gene expression dynamics in primary cells stimulated with either ACTH or AngII is limited to a handful of genes.

RNA sequencing provides a transcriptome-wide view of which processes are activated and repressed during steroidogenesis. Here, we performed an RNA-seq time series on primary human adrenocortical cells stimulated with ACTH or AngII and also on H295R using forskolin or AngII. We found that the ligand-induced changes in gene expression in adult adrenocortical primary cultures involved many of the same genes and similar timing, but different response magnitudes. While stimulus-induced expression changes in H295R cells were recapitulated by primary cells, there were also expression changes unique to primary cells. We provide a web application allowing investigators to explore their genes of interest (https://raysinensis.shinyapps.io/steroidogenesis/ accessed on 20 August 2021).

## 2. Materials and Methods

### 2.1. Cell Culture

Primary human adrenal cells were isolated and cultured as described previously [26,27]. Briefly, a portion of an individual adrenal gland was minced and dissociated into a single-cell suspension by repeated exposure of the tissue fragments to DMEM/F12 medium containing 1 mg/mL collagenase dispase and 0.25 mg/mL DNaseI (Hoffmann-La Roche Ltd., Basel, Switzerland). Four 1 h cycles of digestion at 37 °C and mechanical dispersion were performed. Cells were collected between each digestion and combined prior to storage at −150 °C in freezing media containing 50% growth medium, 40% CCS (or fetal bovine serum), and 10% DMSO. Thawed cells were plated at a density of 20,000 cells/well (48 well dish) in growth medium and grown to 60% confluence, and then starved in low serum medium for 18 h prior to treatment with either 10 nM AngII or 10 nM ACTH for the indicated time points. Normal adrenals were collected through the Gift of Life Recovery of Cadaver Adrenal Tissue and Plasma for Research/Gift of Life Michigan (GOLM), which allows for the collection of deceased donor adrenals through the existing organ donation program. IRB approval HUM00069665. For this IRB, the Human Subjects Research falls under Exemption 4. The study was conducted according to the guidelines of the Declaration of Helsinki.

H295R cells were cultured in complete media containing DMEM:F12 (Gibco, Grand Island, KY, USA; 11320-033 ) with 10% Hyclone Cosmic Calf Serum (GE Healthcare, USA; SH30087.03) and 1% ITS+ Premix (Corning, Grand Island, NY, USA; 354352). On the first day, 500,000 H295R cells were plated in complete media. On the second day, cells were switched to low sera media (DMEM:F12 with 0.1% Cosmic Calf Serum, 1% ITS). On the third and fourth days, cells were treated with 10 nM AngII or 10 uM forskolin at different times and simultaneous collection on day 4. The full time course experiment was performed in duplicate. 

### 2.2. RNA-Seq Library Construction

Primary adrenal cell RNA was collected using the RNEasy MiniPrep Plus kit (Qiagen, Hilden, Germany; 74104) following the manufacturer’s instructions. PolyA-containing RNA was collected for each sample from approximately 65 ng total RNA input using one round of the Lexogen Poly(A) selection kit V1.5 (Lexogen GmbH, Vienna, Austria; 157.96) following the manufacturer’s instructions, and then used as input in downstream RNA-seq library preparation via the KAPA RNA HyperPrep kit (KAPA Biosystems, Wilmington, NC, USA; KK8541) and dual index adapters (KAPA Biosystems, Wilminton, NC, USA; KK8722). Final libraries were amplified with 15 cycles of PCR. 

H295R cells were collected in TRIzol and RNA was isolated using Zymo Research Direct-Zol Miniprep Plus kit (Zymo Research, Irvine, CA, USA; R2072) following the manufacturer’s instructions with on-column DNase I digestion. An amount of 500 ng total RNA was used as input into ribosomal RNA depletion, followed by RNA-seq library preparation using KAPA Biosystems KAPA RNA HyperPrep kit with RiboErase (KAPA Biosystems, Wilmington, NC, USA; KK8561) and dual index adapters. Final libraries were amplified with 10 cycles of PCR.

Sequencing was performed on the NovaSeq 6000 (Illumina, San Diego, CA, USA) for 2 × 150 bp paired-end (PE) reads at the Genomics Shared Resource at the University Of Colorado Cancer Center (Aurora, CO, USA).

### 2.3. Real Time Quantitative PCR

Validation of RNA-seq was performed on 12 h and 24 h primary culture samples using real-time quantitative PCR. Reverse transcription was performed using 0.1 ng of RNA input for the iScript kit (Bio-Rad, Hercules, CA, USA) and qPCR using iTaq Universal SYBR Green Supermix (Bio-Rad, Hercules, CA, USA) on a CFX 384 qPCR instrument (Bio-Rad, Hercules, CA, USA). Analysis of qPCR was performed using the delta-delta Cq method normalizing to the average values of RPS20 and RPS13A within the sample and then time-matched basal media sample.

### 2.4. RNA-Seq Data Analysis

The code used for data analysis is available at https://github.com/mukherjeelab/primary-h295r-comparison (accessed on 26 Augusts 2021). Read mapping and quantification via salmon [28] used settings “-l-A-allowDovetail-validateMappings” and a custom salmon index containing Gencode v26 precursor and mature transcripts and ERCC spike-ins as was used previously [29]. Next, salmon quantification tables were imported into edgeR framework via tximport for differential expression analysis using generalized linear models. Differentially expressed genes were determined at FDR < 0.001 cutoff. For forskolin stimulation of H295R cells, differentially expressed genes were grouped by k-means clustering, with the number of clusters optimized by the elbow method. Quantification of transcript-per-million (TPM) and fold change values are presented in an interactive R shiny data browser, at https://raysinensis.shinyapps.io/steroidogenesis/ (accessed on 20 August 2021). For H295R stimulation, fold change values are calculated by normalizing gene quantification against zero-hour untreated condition. For primary cells, stimulated samples were normalized against basal condition of the corresponding time point. Code for running the data browser locally can be found in the “shiny” subdirectory of the GitHub repository.

### 2.5. Gene Set Enrichment Analysis

For each treatment condition, normalized enrichment scores (NES) were calculated by Gene Set Enrichment Analysis against MSigDB hallmark gene sets or AngII-H295R temporal and amplitude clustering gene sets as reported previously, using R packages clusterProfiler. For visualization in heatmap form via ComplexHeatmap, hallmark gene sets with no significant (*q* ≤ 0.05) enrichment or depletion in any condition were excluded, and remaining gene sets were ordered by fold change compared to basal culture. 

## 3. Results

### 3.1. Expression Responses of Known Steroidogenic Genes in Primary Human Adrenocortical Cells Stimulated with ACTH or AngII

**Figure 1:**
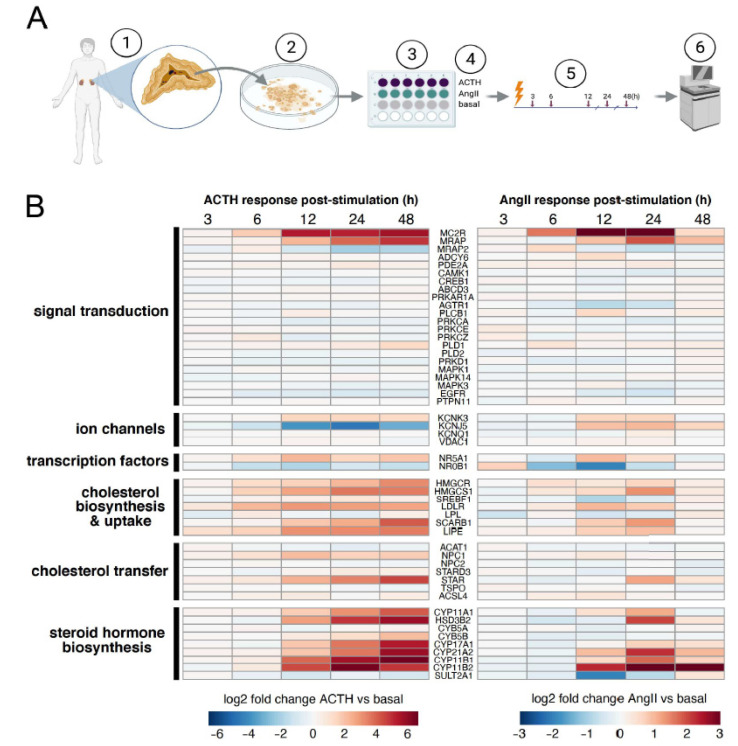
Steroidogenic expression responses of primary human adrenocortical cells to adrenocorticotropic hormone (ACTH) or angiotensin II (AngII). (**A**) Primary cell culture schema: (1) Adrenal gland was coincidentally removed from a deceased human kidney donor. (2) Cortical cells were isolated, dissociated and then (3) plated in triplicate and allowed to reach confluence before being (4) stimulated with ACTH (purple), AngII (green), or left untreated for basal (gray). (5) RNA was purified from cells stimulated for 3, 6, 12, 24, and 48 h. (6) RNA sequencing libraries were prepared after poly(A) selection. (**B**) Comparison of normalized responses for ACTH (left) and AngII (right). Heatmap of log_2_ fold changes in RNA expression between stimulated and unstimulated cells. Red denotes increased expression while blue shows decreased expression. Genes are categorized by pathway.

To understand the transcriptome-wide steroidogenic response of human adrenocortical cells, we performed RNA-seq on human adult adrenocortical cells stimulated with either AngII or ACTH (Figure 1A). Primary adult adrenocortical cells were isolated from human adrenal glands removed from kidneys marked for transplant donation [22,30].

We characterized the timing of stimulation-induced gene expression responses by assaying five different post-stimulation time points (3, 6, 12, 24, and 48 h), in triplicate. All replicates were highly correlated (average R ~0.97, Supplementary Figure S1 and Table S2). To confirm the quality of our data, we curated a list of genes with established roles in aldosterone and cortisol production and examined how they responded to stimulation by ACTH or AngII. Indeed, many of the same steroidogenic transcripts were induced by both ligands (Figure 1B and Supplementary Table S2). For example, several genes pertaining to steroid hormone biosynthesis (bottom group) were induced by both ACTH and AngII (heatmap, red). This included *CYP11B1*, which encodes the enzyme responsible for the final step of cortisol synthesis. Additionally, there was also induction of *CYP11B2*, which encodes aldosterone synthase which carries out the last step of aldosterone synthesis [30]. While both ligands induced the expression of *CYP11B1* and *CYP11B2*, *CYP11B1* exhibited preferential induction in response to ACTH compared to AngII. We also identified discordant responses, such as *KCNJ5*, a voltage-gated K+ channel, that was substantially repressed by ACTH stimulation and modestly induced by AngII stimulation. While the majority of genes induced by AngII were also induced by ACTH, the magnitude of the AngII-induced changes was less than the magnitude of ACTH-induced expression changes (see heatmap scale Figure 1A). Altogether, we found that many genes known to be involved in aldosterone and cortisol synthesis were responsive to ACTH and AngII, indicating that our data were of high quality and recapitulated expected behavior.

### 3.2. Transcriptome-Wide Comparison of Expression Response between ACTH and AngII in Primary Adrenocortical Cells

**Figure 2:**
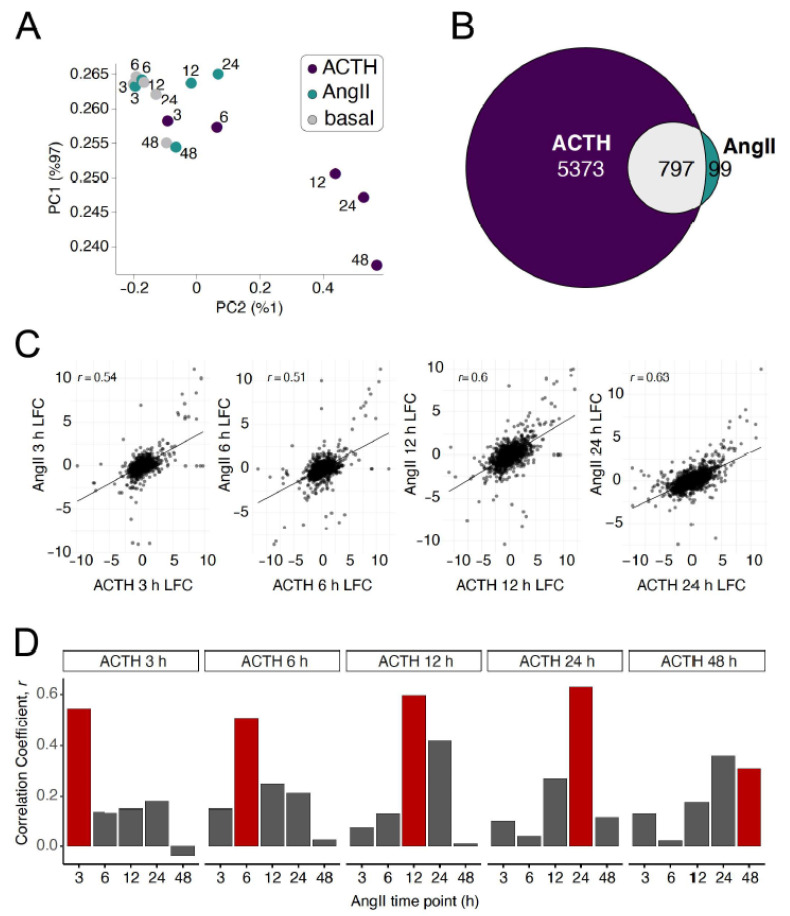
Comparison of transcriptome-wide expression response between ACTH and Ang II in primary adrenocortical cells (**A**) Principal components (PC) analysis of all stimulation treatments and time points mean expression of replicates for all expressed genes. Points are colored by stimulation treatment; ACTH (purple), AngII (green), or left untreated for basal (gray). (**B**) Euler diagram of the genes that were differentially expressed at any time point in response to ACTH (purple) compared to AngII (green) and their intersection (gray). (**C**) Scatterplot of expression changes induced by ACTH (X axis) or AngII (Y axis) for matching time points. (**D**) Barplot showing the correlation of gene expression changes induced by AngII and ACTH by each time point. The red bars denote the matched time point for either stimulation treatment.

We next focused on characterizing the transcriptome-wide similarities and differences of primary cells stimulated with ACTH or AngII. We performed principal components analysis (PCA) using all expressed genes to compare the differences between treatments. Relative to untreated cells (basal), ACTH-stimulated cells resulted in larger transcriptomic differences than cells stimulated with AngII (Figure 2A).

We then identified genes that exhibited statistically significant differential expression due to stimulation with ACTH or AngII. We found 6170 genes differentially expressed at one or more time points due to ACTH stimulation. However, AngII stimulation resulted in only 896 differentially expressed genes. The vast majority of AngII-regulated genes were also regulated by ACTH (*n* = 797). Further, 5373 differentially expressed genes were unique to the ACTH stimulation while only 99 genes were unique to AngII stimulation (Figure 2B). Based on the relatively few genes unique to AngII induction, the overwhelming overlap with the ACTH-regulated genes set, and general weakness of AngII induction from the curated list of genes, we next examined whether the differences between the ACTH and AngII response was qualitative or quantitative. First, we compared the degree of gene expression changes induced by ACTH versus AngII using the union of all differentially expressed genes (*n* = 6269) for corresponding stimulation time points (Figure 2C). Apart from the 48 h time point comparison (Supplementary Figure S2), the changes in expression were correlated (R: 0.51–0.63). These results suggested that the expression response of ACTH and AngII stimulation were similar, and the differences could largely be explained by the magnitude of the change in expression rather than different genes changing upon stimulation. We extended the correlation analysis to compare each time point ACTH stimulation compared to each time point of AngII stimulation and found that the similarity between AngII- and ACTH-induced expression changes were highest for matching time points (Figure 2D, red bars). These data demonstrate that the dynamics of ACTH-induced expression changes were similar to the dynamics of AngII-induced expression changes in primary adrenocortical cells.

### 3.3. ACTH- and AngII-Regulated Pathways in Primary Adrenocortical Cells

**Figure 3:**
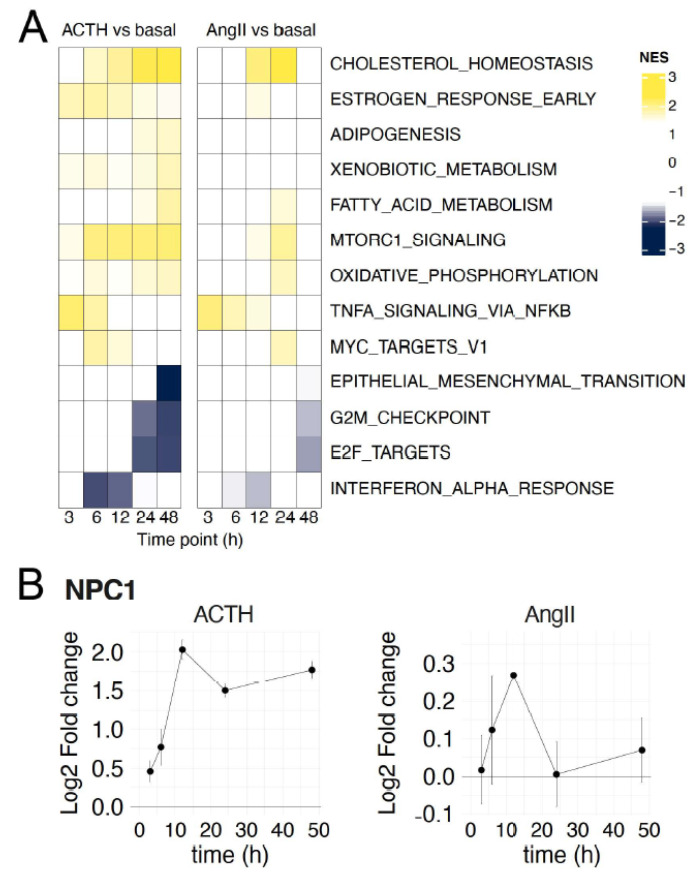
Comparison of ACTH- and AngII-induced gene expression hallmarks in primary cells. (**A**) For each treatment condition, normalized enrichment scores (NES) were calculated by Gene Set Enrichment Analysis against Molecular Signatures Database (MSigDB) hallmark gene sets, ordering genes by fold change compared to basal culture. Yellow denotes increased enrichment while blue shows depletion. Hallmark gene sets with no significant (*q* ≤ 0.05) enrichment or depletion in any condition were excluded from the plot. (**B**) Line plot of log_2_ fold change in expression of *NPC1* upon treatment with ACTH (left) and AngII (right). Error bars are standard deviation.

To better understand which gene pathways were up or downregulated in response to ACTH or AngII, we performed Gene Set Enrichment Analysis (GSEA). Specifically, we used the “hallmark gene sets”, which were curated to represent distinct and diverse biological processes [31]. Generally, both ligands upregulated or downregulated the same pathways (Figure 3A). For example, the hallmark set for cholesterol homeostasis was upregulated by both ligands, consistent with previous reports indicating that stimulation promotes cholesterol biosynthesis, cholesterol ester uptake, and cholesterol transport [32,33,34,35]. Interestingly, adipogenesis and xenobiotic metabolism were enriched by ACTH stimulation, but not by AngII stimulation. A key gene differentially regulated uniquely by ACTH stimulation was *NPC1*, which is involved in intracellular trafficking of cholesterol [36]. *NPC1* was highly induced by ACTH stimulation in primary cells, while much less responsive to AngII stimulation (Figure 3B). To exclude the possibility that these changes were technical artifacts, we performed qRT-PCR validation of *NPC1* for the 12 h and 24 h time points. These results validated that *NPC1* was induced by ACTH, but not AngII (Supplementary Figure S3). Overall, the vast majority of pathways exhibited concordant expression changes for both ACTH and AngII stimulation.

### 3.4. Similarity in Expression Changes and Temporal Response between Primary Adrenocortical Cells and H295R Cells

**Figure 4:**
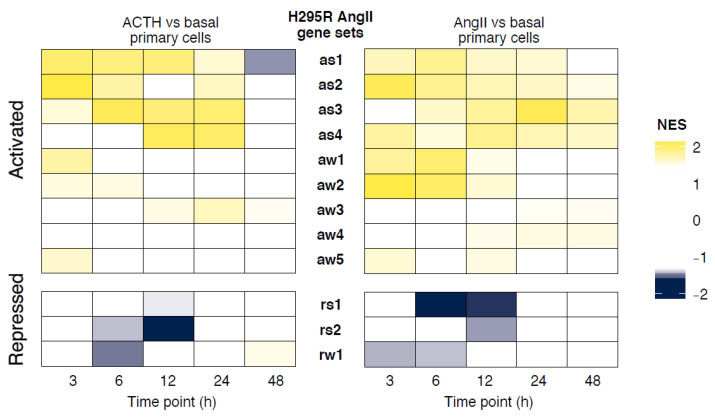
Projection of AngII-induced H295R differential expression gene clusters onto ACTH- and AngII-induced primary cell expression changes. For each primary cell treatment time point (ACTH left, AngII right), normalized enrichment scores (NES) were calculated by Gene Set Enrichment Analysis against AngII-H295R gene sets determined by temporal and amplitude clustering as reported previously, ordering genes by fold change compared to basal culture. Yellow denotes enrichment while blue shows depletion. H295R AngII gene sets were named by their profile for: direction-activation (a) or repression (r), magnitude-strong (s) or weak (w), and timing-numbered from early to late peak response (1 earliest to peak).

The H295R cell line, which was derived from an adrenocortical carcinoma [13,14,15,16], is the main cell culture model to study human adrenocortical steroidogenesis. To understand how this immortalized cell line compared to normal adrenocortical cells, we asked how well the changes induced by steroidogenic stimuli were recapitulated in primary cells. We previously performed an RNA-seq time course of H295R cells stimulated with AngII and we established 12 groups of genes exhibiting distinct temporal response profiles [25]. Likewise, we stimulated H295R cells using forskolin, an activator of adenylyl cyclase, which resulted in five temporal response profiles. The forskolin stimulation time course consisted of only six time points, as opposed to 12, which likely contributed to the fewer distinct response groups. Similar to the analysis of gene sets (see Figure 3), we performed GSEA using the experimentally defined H295R stimulation gene sets to test whether they were up- or downregulated in primary cells stimulated with ACTH or AngII. Indeed, we found that clusters containing genes activated by forskolin and AngII in H295R cells were also upregulated in response to ACTH and AngII, respectively, in primary cells (Figure 4 and Supplementary Figure S4). Likewise, genes repressed in H295R cell responses were also downregulated in primary cell responses. Interestingly, the early activated H295R gene sets were more enriched in early time points of primary cell response and late activated gene sets were more enriched in the later time points of primary cell response (Figure 4). These results demonstrated that the direction and timing of expression changes induced by steroidogenic stimuli in H295R cells were largely recapitulated in primary adrenocortical cells.

### 3.5. Gene-Level Comparison between Primary and H295R Cell Responses

**Figure 5:**
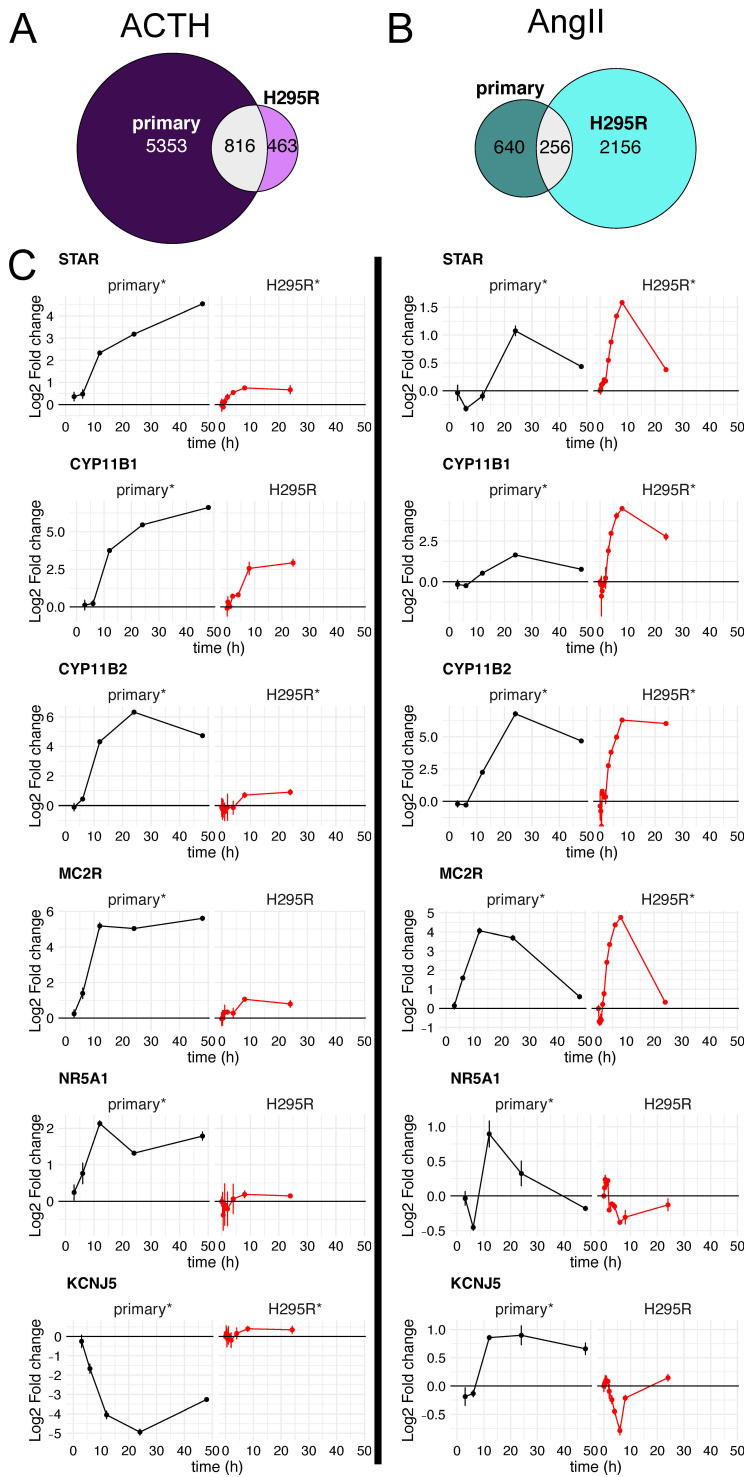
Comparison of stimulus-induced expression changes between primary adrenocortical cells and H295R cells. (**A**) Euler diagram of differentially expressed genes in primary cells stimulated with ACTH (dark purple) as compared to differentially expressed genes in H295R cells stimulated with forskolin (light purple) and their intersection (gray). (**B**) Euler diagram of differentially expressed genes in primary cells stimulated with AngII (dark green) as compared to differentially expressed genes in H295R cells stimulated with AngII (light green) and their intersection (gray). (**C**) Line plot of log_2_ fold change in expression upon treatment with ACTH or forskolin (left) and AngII (right). For primary cells, log_2_ fold changes were calculated versus basal media at matching times, whereas for H295R cells, log_2_ fold changes were calculated versus unstimulated cells. Black lines represent log_2_ fold changes of primary cells and red lines represent log2 fold changes of H295R cells. Error bars are standard error. An asterisk by primary or H295R indicates whether the gene exhibited statistically significant differential expression (see methods for details).

For ACTH-stimulated (left) and AngII-stimulated (right) cells, we compared differentially expressed gene sets by cell type (Figure 5A and Supplementary Table S3). Upon ACTH stimulation, 6169 genes changed in expression for primary cells, while forskolin-induced changes in 1279 genes for H295R cells (Figure 5A). The vast majority of the forskolin-regulated H295R changes were recapitulated in primary cells stimulated with ACTH (*n* = 816). However, a large set of expression changes (*n* = 5353) were unique to primary cells. In contrast, AngII stimulation of H295R cells (*n* = 2412) resulted in almost three fold more differentially expressed genes than AngII stimulation of primary cells (*n* = 896). This is consistent with our earlier observation that fewer genes were differentially expressed in AngII-stimulated primary cells (Figure 2B). Furthermore, when we compare the AngII-regulated genes to the more robust ACTH response in primary cells, we found the changes were generally concordant (Figure 4-left). We conclude that stimulation-induced changes identified in H295R cells were largely recapitulated in the primary human adrenocortical cells.

In the following section, we examined biologically relevant gene expression responses to ACTH- and AngII-induced expression changes in primary cells, as well as, forskolin and AngII-induced expression changes in H295R cells (Figure 5C, see Supplementary Figure S5 for actual expression levels in transcripts per million (TPM)).

- *STAR* is a key steroidogenic protein that facilitates the transport of cholesterol from the outer mitochondrial membrane to the inner mitochondrial membrane, which is the rate-limiting step of adrenocortical steroidogenesis [37]. *STAR* was significantly induced in each cell type and by both ligand pathways (Figure 5C-top). However, the ~16-fold induction in primary cells upon ACTH treatment was substantially more than ~2-fold induction by forskolin in H295R cells or either primary or H295R cells treated with AngII. Basal *STAR* expression was ~5-fold higher in primary cultures (~800 TPM) than H295R cells (~150 TPM).
- The P450 cytochrome enzymes are important players in steroid hormone production. Specifically, *CYP11B1* and *CYP11B2* are responsible for the final step of cortisol and aldosterone synthesis, respectively. Both *CYP11B1* and *CYP11B2* were significantly induced by all ligands in primary cells, with *CYP11B1* induction by AngII being the weakest as expected (Figure 5C). The largest outlier was the very modest ~2-fold induction of *CYP11B2* by forskolin in H295R cells; all other combinations of ligands and cells resulted in at least 60-fold induction of *CYP11B2*. The induction of CYP11B2 by ACTH and AngII in primary cells was validated using qRT-PCR (Supplementary Figure S3).
- *MC2R* and *MRAP* are both critical components of receptor-mediated signaling by ACTH [38]. These genes are both expressed at low levels in H295R cells (avg TPM of 5, 2, respectively) and have much higher expression in primary cells (avg TPM of 40, 60, respectively). This is consistent with the insensitivity of H295R cells to ACTH. Regardless, we did indeed detect statistically significant changes in gene expression for *MC2R* for primary cells stimulated with ACTH or AngII as described previously [39]. Similar to *CYP11B2*, forskolin stimulation of H295R cells resulted in only a modest increase in *MC2R*, which was at least 16-fold induced in all other cell type ligand combinations.
- *NR5A1*, also known as *SF-1*, is a transcriptional activator for several P450 cytochrome enzymes and *STAR* [40]. This key regulator showed significant induction for the ACTH treated primary cells (~4-fold) only very modest induction in AngII treated primary cells (~1.9-fold). However, there were no significant changes in *NR5A1* mRNA levels in H295R cells, even though the basal expression level of *NR5A1* is similar in both primary and H295R cells (~25 TPM).
- *KCNJ5* encodes an inward rectifying K+ channel crucial for the influx of Ca+ that precedes the PKC signal transduction pathway and transcriptional upregulation of pro-steroidogenic genes [41]. Both ACTH and AngII also lead to differential expression of *KCNJ5* in primary cells, but not in H295R cells. However, *KCNJ5* exhibited a discordant response in primary cells between ACTH and AngII stimulation. While we were not surprised to see *KCNJ5* induction upon AngII stimulation in primary cells because it is predominantly expressed in *glomerulosa* cells, we were surprised to see this gene repressed upon ACTH stimulation in the same cell type. The repression of *KCNJ5* by ACTH, but not AngII in primar cells was validated using qRT-PCR (Supplementary Figure S3)

Together, these data demonstrate that expression changes induced in H295R cells by steroidogenic stimuli were also induced in primary human adrenocortical cells. However, there are expression changes induced in primary adrenocortical cells, particularly for ACTH stimulation, that are not recapitulated in H295R cells.

## 4. Discussion

One of the goals of this study was to identify similarities and differences in gene expression responses for ACTH and AngII stimulation in primary human adrenocortical cells. We found that the ligand-induced changes in gene expression largely involved the same genes and similar kinetics. However, the magnitude of expression responses was substantially larger for primary cells stimulated with ACTH compared to AngII. This raises the question of whether the difference in magnitude is because the following possibilities: (1) the difference in concentrations of ACTH and AngII, (2) ACTH is more stable than AngII and results in repeated or longer duration of stimulation, (3) the fraction of cells from each zone in the pool of primary cells, and relatedly, (4) the fraction of aldosterone-producing cells in the population. AT1 receptors that recognize AngII have much higher expression in *zona glomerulosa* cells compared to *zona fasciculata* or *reticularis* [42]. Furthermore, only a subset of adult *zona glomerulosa* cells, often clustered together, appear to produce aldosterone based on positive staining for *CYP11B2* [43]. Indeed, the *ACTH* receptor *MC2R* and its required accessory *MRAP* are expressed in both the zona glomerulosa and zona fasciculata of humans and rats [44,45,46]. Acute ACTH stimulation is known to increase aldosterone synthesis initially and then synthesis decreases after a few days [46]. Therefore, the stronger magnitude of the ACTH response in the mixed population of primary cells may be caused by a higher percentage of cells stimulated by ACTH than by AngII. Experiments that either resolve or minimize cellular heterogeneity, such as single-cell approaches and/or purified populations of zone-specific cells, will be crucial to distinguish between the aforementioned possibilities.

ACTH and AngII stimulation of primary cells caused similar expression responses for most genes with respect to timing and direction. Nevertheless, we identified steroidogenesis associated genes with discordant responses as well. *NPC1* is found on the membranes of endosomes and lysosomes, and transports cholesterol to the mitochondria. This pathway utilizes lipids bound to the plasma membrane to contribute to the free pool of cholesterol. *NPC1* is not necessary for steroidogenesis because the cell can also synthesize its own cholesterol under control of ACTH [35]. We observed that ACTH stimulation led to much greater induction of *NPC1* than AngII stimulation. Therefore, depending on the specific response to stimulation, pools of cholesterol may be derived from different sources in the cell in a ligand-dependent manner. *KCNJ5* encodes the potassium channel *GIRK4*, which is a G protein-coupled inwardly rectifying potassium channel predominantly expressed in the *zona glomerulosa* [47,48]. Accumulation of K^+^ can facilitate depolarization and influx of Ca^+^ and downstream signal transduction. Many aldosterone-producing adenomas (APA) have mutations in *KCNJ5* that cause primary aldosteronism and make cells hyperresponsive to ACTH [12,49]. We observed that in response to ACTH primary cells dramatically downregulate *KCNJ5*, which may limit the extent of subsequent stimulation. It will be interesting to determine whether this potential mechanism is lost in *KCNJ5* mutant APAs. A major caveat is that we only measure RNA levels and are blind to both translational and post-translational regulation. Indeed, comparing transcriptomic changes to proteomic changes upon Ang II stimulation [50] would provide a starting point for evaluating translational and post-translational contributions. Nevertheless, these two examples highlight potentially important differences between ACTH and AngII stimulation and the types of hypotheses that can now be generated using these data. 

The steroidogenic response to stimulation may be more comparable across cell types and ligands with additional dose optimization. We used 10 nM AngII, 10 nM ACTH, and 10 μM forskolin, which are typical dosages for the treatment of human adrenocortical cells. However, in the adrenal tissue of organisms, the amount of ligand varies substantially by circadian and ultradian rhythms [51]. Additionally, the responsiveness of any given carcinoma cell depends on culture conditions [23]. Comparison across studies and optimization of ligand dosage is additionally complicated in treatment of primary cells given the variability in the fraction of cells expressing relevant receptor and required components. Additionally, forskolin treatment of H295R cells resulted in relatively modest induction of several steroidogenic genes compared to primary cultures stimulated with ACTH. It is unclear whether this is caused by differences between ACTH versus forskolin signaling, or inherent differences between primary cells and H295R cells. These differences may include the low expression of *MC2R-MRAP* and the activating mutation in *CTNNB1* found in this cell line. Additional experiments comparing forskolin and ACTH in primary cells and H295RA variant cell line, which expresses *MRAP* [17], would be informative.

Another goal of this study was to evaluate the immortalized H295R adrenocortical carcinoma cell line as a model for normal steroidogenesis with respect to gene expression responses. Since the primary cells and H295R cells utilized different library preparation, we focused our analysis on fold change and differential expression comparisons within the samples to minimize potential bias. Nevertheless, caution should be exercised when interpreting non-polyadenylated RNAs that will not be represented in the poly(A) selected primary cell samples. Most of the stimuli-induced changes in H295R also occurred in primary adrenocortical cells. This was particularly true of the H295R AngII stimulation response, which exhibited striking similarity in direction and timing with both ACTH and AngII stimulation response in primary cells. Perhaps not surprisingly, these data show that AngII stimulation of H295R cells is more physiologically relevant than stimulation of H295R cells with forskolin. However, we also identified many genes exhibiting ACTH-induced changes in primary cells that were either unchanged or modestly regulated in H295R cells. *NR5A1* is a transcription factor for several P450 cytochrome enzymes and *STAR*, making it a key player in the transcriptional activation necessary for timely production of hormones. Indeed, some of these differences could be due to induction of *NR5A1* in primary cells but not H295R cells. Another caveat to this study is the use of primary adrenocortical cells from a single donor. Although primary cell cultures are a valuable tool in this field, there is limited access to fresh donor adrenal tissue and not all primary cells isolated from individual donors pass the rigorous screening process [21,22,30]. As such, we cannot account for variability in expression response between individuals and do not know whether the expression responses observed in these primary cells occur in all healthy adult adrenals. Nevertheless, these data indicate that while AngII-induced changes in H295R are physiologically relevant, they do not capture all the potential physiologically relevant changes in primary cell cultures. Thus, H295R cells are a faithful yet somewhat incomplete model for AngII-mediated aldosterone synthesis. Investigators can easily evaluate whether H295R is an appropriate model for a particular gene of interest using this webtool (https://raysinensis.shinyapps.io/steroidogenesis/ accessed on 20 August 2021) to explore the data.

While primary adrenocortical cell cultures address some of the gaps in physiology lost in the H295R system, it too may provide an incomplete picture of steroidogenesis. The organization of the cortical zones and their corresponding expression profiles rely heavily on paracrine signaling [52,53,54]. However, we prepared primary cells as a suspension culture. Therefore, we lost information about the transcriptome spatially and we also removed the physiologically relevant environment created by neighboring cells. This bulk analysis may miss intricate and important pathways where paracrine interactions are called into play.

## Data Availability

RNA-sequencing data can be accessed at the sequence read archive using accession: GSE181158.

## Supplementary Materials

The following are available online at https://www.mdpi.com/article/10.3390/cells10092376/s1, Figure S1: Correlation across primary cell RNA-seq replicates; Figure S2: Comparison of expression changes induced by ACTH and AngII; Figure S3: qRT-PCR validation of gene expression changes detected by RNA-seq; Figure S4: Projection of forskolin-induced H295R differential expression gene clusters onto ACTH- and AngII-induced primary cell expression changes; Figure S5: Comparison of expression responses between primary adrenocortical cells and H295R cells by transcripts per million; Table S1: Stimulus-induced fold changes primary and H295R experiments; Table S2: Expression levels of all genes in all samples in transcripts per million; Table S3: List of differentially expressed genes in primary vs. H295R cells by stimulation.
