# Supplementary material for: Transcriptomic Response Dynamics of Human Primary and Immortalized Adrenocortical Cells to Steroidogenic Stimuli
Source: Cells. 2021 Sep 9;10(9):2376. doi: 10.3390/cells10092376 (PMC8466536; doi:10.3390/cells10092376)
Supplement: Supplementary file 1 [file cells-10-02376-s001.zip › Supplementary materials.pdf]

cells

MDPI

SUPPLEMENTARY MATERIALS

Transcriptomic Response Dynamics of Human Primary and Immortalized Adrenocortical Cells to Steroidogenic Stimuli

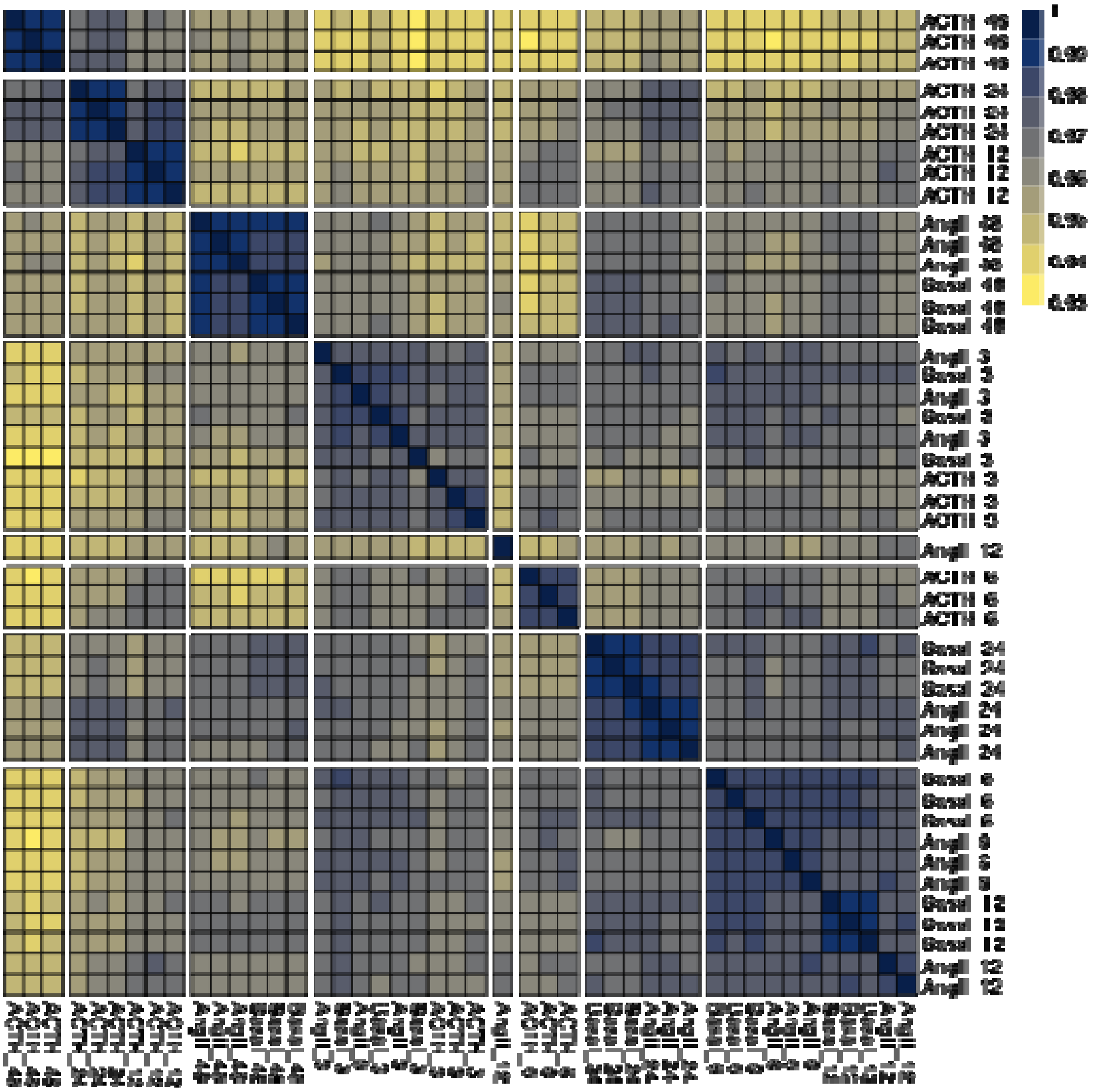

Figure S1. Correlation across primary cell RNA-seq replicates. Heatmap of Spearman correlation coefficients for expressed genes.

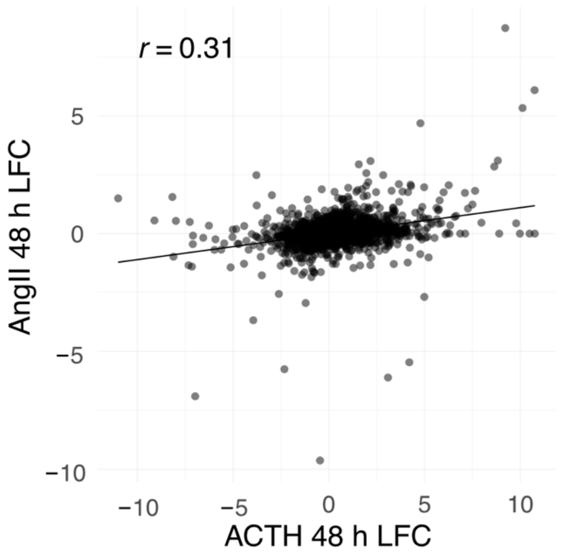

Figure S2. Comparison of expression changes induced by ACTH and AngII. Scatterplot of expression changes induced by ACTH (*x*-axis) or AngII (*y*-axis) for matching 48 h stimulation.

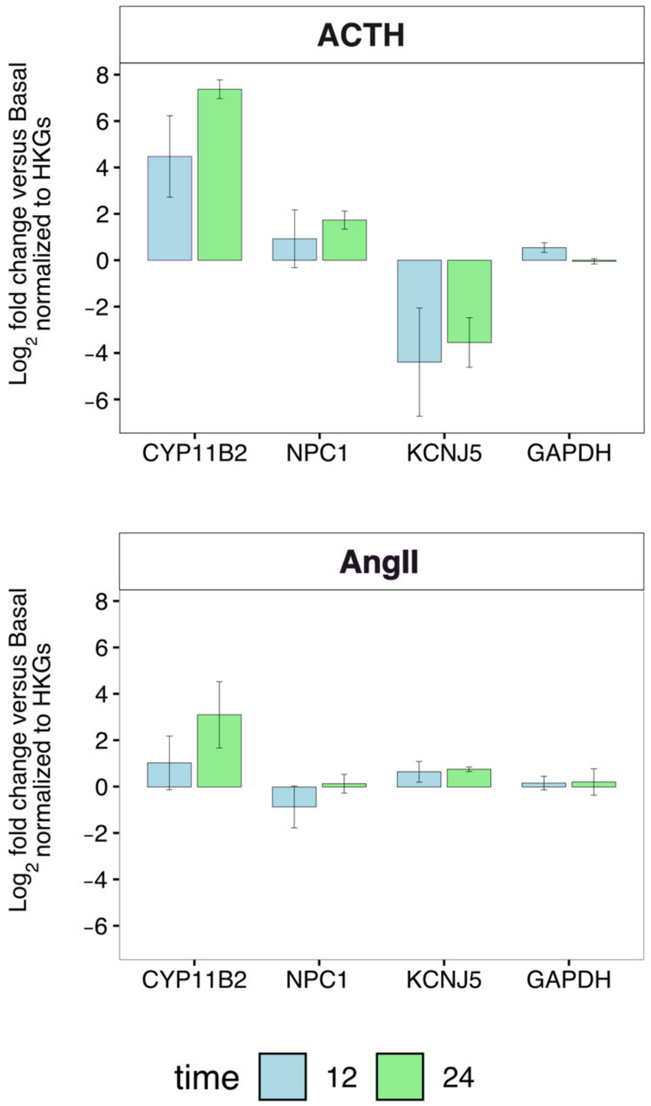

Figure S3. qRT-PCR validation of gene expression changes detected by RNA-seq. Barplot of change in expression of gene indicated in primary cells treated with ACTH (top) or AngII (bottom) compared to Basal at 12 and 24 h post-stimulation. All gene expression measurements were normalized to the average of two housekeeping genes (RPL13A and RPS20). Error bar represents the standard deviation of 3 independent cell treatment replicates.

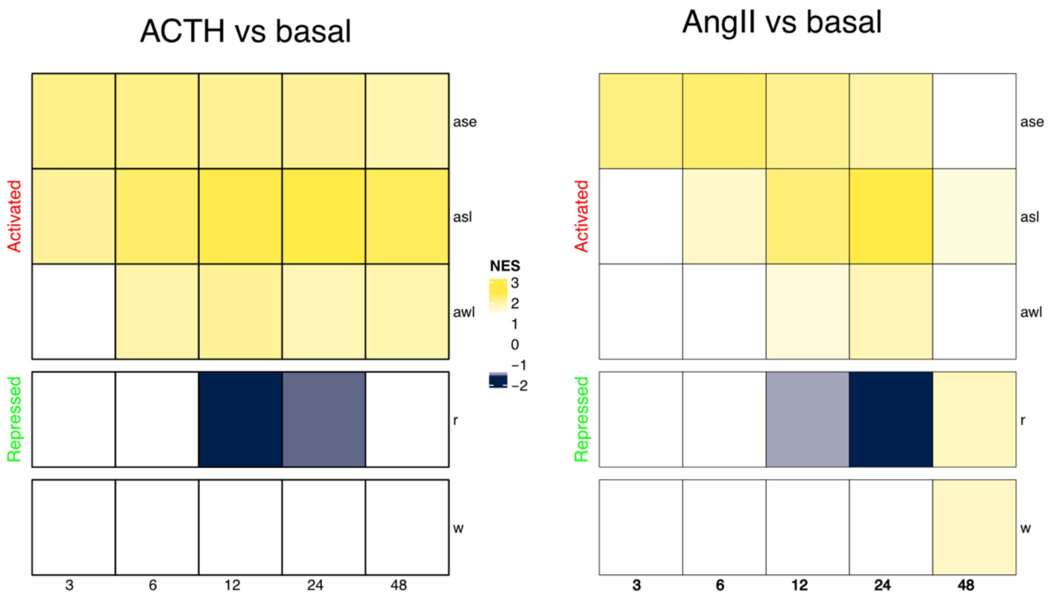

Figure S4. Projection of Forskolin-induced H295R differential expression gene clusters onto ACTH- and AngII-induced primary cell expression changes. For each primary cell treatment time point (ACTH left, AngII right), normalized enrichment scores (NES) were calculated by Gene Set Enrichment Analysis against Forskolin-H295R gene sets determined by temporal and amplitude clustering as reported previously, ordering genes by fold change compared to basal culture. H295R Forskolin gene sets were named by their profile for: direction - activation (a) or repression (r), magnitude - strong (s) or weak (w), and timing - numbered from early to late peak response (1 earliest to peak).

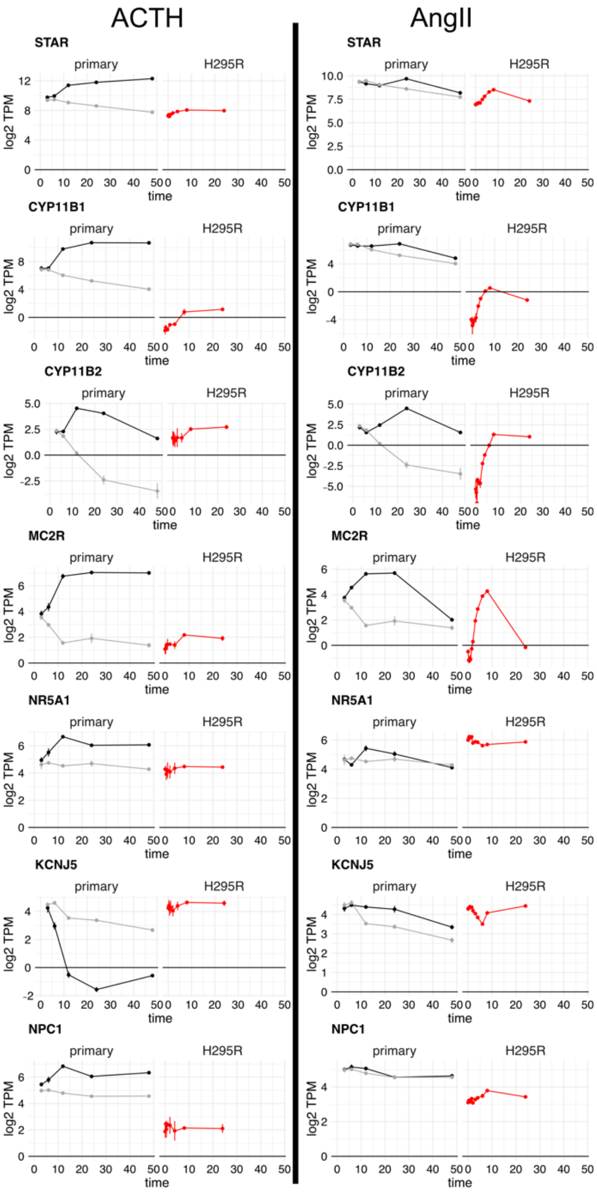

Figure S5. Comparison of expression responses between primary adrenocortical cells and H295R cells by Transcripts Per Million. (A) Line plot of expression levels reported in the $\log_2$ of Transcripts Per Million (TPM) upon treatment with ACTH or forskolin (left) and AngII (right). Black lines represent stimulated primary cells, gray lines represent untreated primary cells, and red lines represent stimulated H295R cells. Error bars are standard error. Error bars are standard error. H295R cells were treated by a reverse time course. Therefore, basal expression at different time points was not measured.

Table S1. Stimulus induced fold changes primary and H295R experiments.

Table S2. Expression levels of all genes in all samples in transcripts per million.

Table S3. List of differentially expressed genes in primary vs. H295R cells by stimulation. Each column contains genes corresponding to each portion of the euler diagram in Figure 5C.

Real-time Quantitative PCR Primers.

Gene	fwd	rev
KCNJ5	CTCAGCTGCATCAGGAAGAGT	GAGGACTGGTGTGAATCGGT
NPC1	TGGGAGCCACTCACGGAT	CGCTCTGTTCCTTTGTATCGC
RPS20	AACAAGCCGCAACGTAAAATC	ACGATCCCACGTCTTAGAACC
RPL13A	CCT GGA GGA GAA GAG GAA AG	TTG AGG ACC TCT GTG TAT TT
GAPDH	CATTGCCCTCAACGACCACTTTGT	TCTACATGGCAACTGTGAGGAGGG
CYP11B2	GGCAGAGGCAGAGATGCTG	CTTGAGTTAGTGTCTCCACCAGGA
